# Elevated Rate of Genome Rearrangements in Radiation-Resistant Bacteria

**DOI:** 10.1534/genetics.116.196154

**Published:** 2017-02-10

**Authors:** Jelena Repar, Fran Supek, Tin Klanjscek, Tobias Warnecke, Ksenija Zahradka, Davor Zahradka

**Affiliations:** * Division of Molecular Biology, Ruder Boskovic Institute, 10000 Zagreb, Croatia; † Medical Research Council London Institute of Medical Sciences, W12 0NN, United Kingdom; ‡ Institute of Clinical Sciences, Faculty of Medicine, Imperial College London, W12 0NN, United Kingdom; § Division of Electronics, Ruder Boskovic Institute, 10000 Zagreb, Croatia; ** Systems Biology Research Unit, European Molecular Biology Laboratory/Centre for Genomic Regulation, 08003 Barcelona, Spain; †† Universitat Pompeu Fabra, 08002 Barcelona, Spain; ‡‡ Division for Marine and Environmental Research, Ruder Boskovic Institute, 10000 Zagreb, Croatia

**Keywords:** *Deinococcus radiodurans*, gamma radiation, genome stability, gene order, synteny

## Abstract

A number of bacterial, archaeal, and eukaryotic species are known for their resistance to ionizing radiation. One of the challenges these species face is a potent environmental source of DNA double-strand breaks, potential drivers of genome structure evolution. Efficient and accurate DNA double-strand break repair systems have been demonstrated in several unrelated radiation-resistant species and are putative adaptations to the DNA damaging environment. Such adaptations are expected to compensate for the genome-destabilizing effect of environmental DNA damage and may be expected to result in a more conserved gene order in radiation-resistant species. However, here we show that rates of genome rearrangements, measured as loss of gene order conservation with time, are higher in radiation-resistant species in multiple, phylogenetically independent groups of bacteria. Comparison of indicators of selection for genome organization between radiation-resistant and phylogenetically matched, nonresistant species argues against tolerance to disruption of genome structure as a strategy for radiation resistance. Interestingly, an important mechanism affecting genome rearrangements in prokaryotes, the symmetrical inversions around the origin of DNA replication, shapes genome structure of both radiation-resistant and nonresistant species. In conclusion, the opposing effects of environmental DNA damage and DNA repair result in elevated rates of genome rearrangements in radiation-resistant bacteria.

ALTHOUGH the ionizing-radiation-resistance phenotype has been found in a number of phylogenetically distant prokaryotes (Cox and Battista 2005; Confalonieri and Sommer 2011), our understanding of the adaptations it entails is incomplete. As one of the most resistant organisms known, the bacterium *Deinococcus radiodurans* has been a model organism for studying radiation resistance (Makarova *et al.* 2001; Cox and Battista 2005; Daly 2009; Confalonieri and Sommer 2011; Slade and Radman 2011; Daly 2012; Krisko and Radman 2013). An important contribution to radiation resistance is thought to come from a DNA double-strand break (DSB) repair system adapted for greater accuracy and efficiency (Battista 1997; Zahradka *et al.* 2006; Slade *et al.* 2009; Confalonieri and Sommer 2011; Slade and Radman 2011). For example, *D. radiodurans* can accurately reassemble its genome after hundreds of DSBs (Dean *et al.* 1966; Minton 1994; Battista 1997). However, DSBs, spontaneous or induced by external factors such as radiation, are potential substrates for genome rearrangement events (*e.g.*, Argueso *et al.* 2008; Repar *et al.* 2010). Therefore, we hypothesized that the opposition between the DSB-inducing environment and the accuracy of DSB repair may result in an evolutionary history of genome rearrangements specific to radiation-resistant prokaryotes.

To check for differences in historical genome rearrangement rates, we first assembled data sets of phylogenetically closely related, completely sequenced prokaryotic genomes that contained both radiation-resistant and nonresistant species. Next, we modeled dynamics of genome rearrangement rates using loss of gene order conservation with time as a proxy. By comparing radiation-resistant *vs.* nonresistant species, we found that in multiple, phylogenetically independent data sets, radiation resistance correlates with elevated rates of genome rearrangements.

## Methods

### Assembling data sets

Radiation-resistant prokaryotes were identified by literature search. The identified taxa were then used to assemble data sets containing phylogenetically closely related radiation-resistant and nonresistant species with completely sequenced genomes. The genome sequences and their annotations were downloaded on October 5, 2014 from the GenBank file-transfer-protocol server (ftp://ftp.ncbi.nih.gov/genbank/genomes/Bacteria/). GenBank species carrying the same name as the ones identified as radiation resistant in the literature were assumed to be radiation resistant.

Radiation-resistant species were used as focal taxa around which data sets containing phylogenetically related species were assembled. The assembly process was based on the crude estimation of 16S ribosomal RNA (rRNA) distances between all the species obtained from GenBank. These distances were estimated by the dnadist program from the PHYLIP 3.6 package (Felsenstein 2005), with a default ClustalX 2.1 (Larkin *et al.* 2007) multiple alignment of 16S rRNA sequences. Lists of 19 different species with the smallest 16S rRNA distance to the focal radiation-resistant species were assembled and manually filtered to exclude species belonging to a different phylum than the radiation-resistant species. Whenever multiple data sets contained the same radiation-resistant species, only the data set containing the largest number of radiation-resistant species was kept for analysis. This process resulted in seven data sets containing two or more radiation-resistant organisms, each data set named after one of the radiation-resistant species it contains.

The data sets and the radiation-resistant species they contain (see Table 1 for references) are, namely, the D_radiodurans data set (*Truepera radiovictrix*, *D. peraridilitoris*, *D. proteolyticus*, *D. deserti*, *D. gobiensis*, *D. geothermalis*, *D. maricopensis*, *D. radiodurans*), the E_faecium data set (*Enterococcus faecium*, *E. faecalis*), the P_arcticum data set (*Psychrobacter arcticum*, *P. cryohalolentis*, *P. PRwf-1*), the K_radiotolerans data set (*Kineococcus radiotolerans*, *Arthrobacter FB24*, *A. aurescens*, *Geodermatophilus obscurus*), the M_radiotolerans data set (*Methylobacterium radiotolerans*, *M. extorquens*), the C_thermalis data set (*Chroococcidiopsis thermalis*, *Arthrospira platensis*), and the T_gammatolerans data set (*Thermococcus gammatolerans*, *Pyrococcus furiosus*, *P. NA2*).

**Table 1:** List of radiation-resistant species in our data sets and references establishing their radiation resistance

Species (GenBank name)	Reference
Deinococcus_deserti_VCD115_uid16691	de Groot *et al.* (2005)
Deinococcus_geothermalis_DSM_11300_uid13423	Ferreira *et al.* (1997)
Deinococcus_gobiensis_I_0_uid46605	Yuan *et al.* (2009)
Deinococcus_maricopensis_DSM_21211_uid43461	Rainey *et al.* (2005)
Deinococcus_peraridilitoris_DSM_19664_uid61295	Rainey *et al.* (2007)
Deinococcus_radiodurans_R1_uid65	Anderson *et al.* (1956)
Deinococcus_proteolyticus_MRP_uid41911	Brooks and Murray (1981)
Truepera_radiovictrix_DSM_17093_uid38371	Albuquerque *et al.* (2005)
Enterococcus_faecalis_OG1RF_uid20843	Anellis *et al.* (1973)
Enterococcus_faecium_DO_uid30627	Anellis *et al.* (1973)
Thermococcus_gammatolerans_EJ3_uid33671	Jolivet *et al.* (2003)
Pyrococcus_furiosus_COM1_uid163827	DiRuggiero *et al.* (1997)
Pyrococcus_NA2_uid65431	IMG database (Markowitz *et al.* 2012)
Psychrobacter_PRwf-1_uid15759	IMG database (Markowitz *et al.* 2012)
Psychrobacter_arcticum_273-4_uid9633	IMG database (Markowitz *et al.* 2012)
Psychrobacter_cryohalolentis_K5_uid13920	IMG database (Markowitz *et al.* 2012)
Methylobacterium_radiotolerans_JCM_2831_uid18817	Nogueira *et al.* (1998)
Methylobacterium_extorquens_PA1_uid18637	Nogueira *et al.* (1998)
Arthrobacter_FB24_uid12640	IMG database (Markowitz *et al.* 2012)
Geodermatophilus_obscurus_DSM_43160_uid29547	Gtari *et al.* (2012)
Kineococcus_radiotolerans_SRS30216_uid10689	Bagwell *et al.* (2008)
Arthrobacter_aurescens_TC1_uid12512	IMG database (Markowitz *et al.* 2012)
Chroococcidiopsis_thermalis_PCC_7203_uid38119	Billi *et al.* (2000)
Arthrospira_platensis_NIES_39_uid42161	Abomohra *et al.* (2016)

IMG, integrated microbial genomes.

References establishing radiation resistance of the radiation-resistant species and GenBank files matched to them are listed in Table 1. All the species in the data sets are listed by their GenBank names in Supplemental Material, Table S2 in File S1. The doses of gamma radiation that reduce survival of a prokaryotic population to 10% (the *D*_10_ values) obtained from the literature for radiation-resistant and nonresistant species are listed in Table S2 in File S1. The *D*_10_ values are not available for all the radiation-resistant species because even though all have been found to survive large doses of ionizing radiation, for some of them no survival curves were measured.

Since our goal was to investigate whether radiation resistance affects genome rearrangement rates, we separated the species within each data set into groups with and without radiation resistance. For a number of species, the information was readily available (Table 1 and Table S2 in File S1). Species of unknown resistance were included in the nonresistant group because (i) it is a conservative approach; and (ii) reporting bias favors reports of radiation resistance, making it more likely that unreported species are nonresistant. The approach is conservative because it increases the statistical power required for a positive result: the resistant species potentially included in the nonresistant group are expected to reduce the differences between the groups, if there are any. Reporting bias favors radiation-resistant species because they are intrinsically more interesting when discussing irradiation, and because they are often isolated from environmental samples after irradiation treatment that kills nonresistant species (*e.g.*, Anderson *et al.* 1956; Jolivet *et al.* 2003; de Groot *et al.* 2005; Rainey *et al.* 2005, 2007; Yuan *et al.* 2009).

When genomes consisted of multiple elements, only the largest genome element was taken into account for all analyses because the largest element is expected to be under the highest selective constraints, with the rest of the genome elements, such as chromids and plasmids, usually carrying accessory, nonessential functions (Harrison *et al.* 2010).

### Genome rearrangement distances

Genome-rearrangement distances between species pairs in each data set were estimated using the differences in the ordering of orthologs between genomes, a commonly used approach (*e.g.*, Suyama and Bork 2001; Tamames 2001; Belda *et al.* 2005; Rocha 2006; Aguileta *et al.* 2014). Orthologs were defined as best bidirectional blast hits (Wolf and Koonin 2012) between the proteins of the two species that did not differ >40% in identity and >20% in length (Rocha 2006). We varied the identity threshold to check the effect of the stringency of ortholog definition on rearrangement distances.

The genome-rearrangement distances were estimated in two ways: (i) as gene order conservation index (GOC, Rocha 2006) by dividing the number of ortholog pairs that were contiguous in both genomes with the total number of shared orthologs, and (ii) as the number of inversions separating the two genomes normalized by the number of shared orthologs. For the latter, we used the GRIMM version 2.01 program (Tesler 2002) to calculate the number of steps of the most parsimonious scenario transforming one genome into another by inversions. The dependence of rearrangement distance on evolutionary time (estimated as 16S rRNA distance), *i.e.*, the dynamic of loss of gene order conservation with time, is referred to as the genome-rearrangement rate throughout the article.

The variability of the ortholog number shared by two species may affect the measures of rearrangement distance. To reduce the effect of the variations in the number of orthologs, we used an average GOC obtained by (i) 100 resamplings of 250 orthologs selected at random from the ortholog set of each pair of genomes (GOC_250_), and (ii) 100 resamplings of 100 orthologs selected at random from the ortholog set (GOC_100_). The same resamples used for calculating GOC_250_ were used to obtain the inversion-rearrangement distances (GRIMM_250_).

### 16S rRNA distance

To estimate the time during which genome rearrangements could have taken place, we used refined 16S rRNA distances between species in each data set. The 16S rRNA sequences were aligned for each data set using SSUAlign version 0.1 (Nawrocki *et al.* 2009), with default settings. To extract the most informative blocks of aligned sequences, the alignments were filtered using Gblocks Server version 0.91b (Castresana 2000) with the settings allowing for smaller final blocks and gap positions within the final blocks. The resulting alignments were then used to calculate pairwise 16S rRNA distances, as well as the maximum likelihood phylogenetic tree, using TreePuzzle 5.2 (Schmidt *et al.* 2002) with the Hasegawa–Kishino–Yano model of substitution (Rocha 2006). The pairwise 16S rRNA distances were used throughout the rest of the article as the estimate of evolutionary time, *i.e.*, time available for the accumulation of rearrangements.

### Modeling the interdependence of genome rearrangements and 16S rRNA distances

The model described by Equation 1 used for the fitting of GOC_250_ *vs.* 16S rRNA distance was taken from Brilli *et al.* (2013), where it was obtained as a variant of the mechanistic model from Rocha (2006) that gave one of the best fits of all the simple (two or less parameter) models.

$$\mathrm{GOC}=f_{i}+\left(\text{1}-f_{i}\right)p^{x}.$$ (1)

The parameter *f_i_* describes the saturation level of the data sets—the portion of the ortholog pairs between which breakpoints are rarely introduced. The parameter *p* describes the rate of gene order conservation loss with time, and depends on the rate of rearrangements within the other portion of ortholog pairs; and *x* represents the 16S rRNA distance. The GOC_250_ *vs.* 16S rRNA distance points for different data sets were pooled and fitted together for the general prokaryotic model in Figure 2A, or fitted separately to the points of each data set (data set models in Figure 2C, parameters given in “separate data sets” part of Table S3 in File S1). Additionally, the model was fitted to three different categories of GOC_250_ *vs.* 16S rRNA distance points defined by radiation resistance (R) or nonresistance (N) of species in the species pair (the categories are R-R, R-N, and N-N). The three different categories of points were fitted in one of two ways: each category fitted independently (parameters given in “six parameters” part of Table S3 in File S1), or with the constraint that parameter *f_i_* be fixed for all the categories (to facilitate the comparison of rates of gene order conservation loss for different categories of points). In the latter case, the parameter *f_i_* was fixed at a value obtained for the whole data set model (*i.e.*, at the value given for “separate data sets” in Table S3 in File S1). These fits are shown in Figure 3 and Figure 4 (parameters given under “four parameter models” in Table S3 in File S1). The rate of decline of the fitted curves depends on the parameter *p* and enables comparisons of rates of genome rearrangement—the steeper the decline, the faster the rate (*e.g.*, Figure 3 and Figure 4). Parameters were fitted using a least-squares method with Nelder–Mead optimization implemented in the MATLAB routine “nmregr” of the DEB tool software (as described in Kooijman 2016). The routine minimizes the sum of squares of differences between simulations and data. Bias-corrected and accelerated bootstrap confidence intervals for parameters were constructed after resampling the data randomly with replacement 10,000 times. The parameter values for different data sets and their confidence intervals are given in Table S3 in File S1.

### Estimating genome stability

The genome-stability index for each organism was defined as the average difference between the measured GOC_250_ (for all the pairwise comparisons in which the organism has participated) and the GOC_250_ predicted by a model (Rocha 2006). In the data set of size *N*, each organism participated in *N* − 1 pairwise comparisons. The model we used to describe the change of GOC_250_ with 16S rRNA distance is given in Equation 1. Permutation tests were used to compare genome stability of radiation-resistant and nonresistant genomes to control for the nonindependence of GOC_250_ *vs.* 16S rRNA distance points (each point represents a pair of species, and each species participates in multiple points).

### Analysis of repetitive sequences in the genomes

We looked at repetitive sequences because they can mislead the bacterial DSB recombinational-repair system into creating genomic rearrangements by using ectopic repeats for the reattachment of DNA strands (Kowalczykowski *et al.* 1994). Repetitive sequences were detected using RepSeek (Achaz *et al.* 2007). Exact repeats can be reported by RepSeek based on (i) preset minimal length of the repeat, or (ii) preset maximal *P*-value of the repeat length. RepSeek assigns a *P*-value to exact repeats based on the length and GC content of the genome. Presetting a maximal *P*-value for the repeat length allows calculation of the minimal length of the statistically significantly long repeat for a genome under observation; repeats with a length above this minimal length are not expected to occur by chance in a random genome of the given length and GC composition. Setting the RepSeek *P*-value threshold for exact repeat detection to 0.001 retrieves minimal repeat lengths of 23–29 bp for the species in our data sets. These lengths roughly correspond to the minimal length of homology necessary for DSB repair by homologous recombination [*e.g.*, minimal efficient processing segment (MEPS) for the *Escherichia coli* RecBCD-dependent DSB repair system is 23–27 bp (Shen and Huang 1986)]. The threshold of 0.001 was chosen based on similar previous observations (Achaz *et al.* 2003, 2007). Hence, we used the *P* < 0.001 setting to detect repeats in the genomes in our data sets. When analyzing only longer exact repeats, we set the lower length limit of repeats to 100 bp, without setting the *P*-value threshold.

The final output of RepSeek included all the pairs of exact repeats (the “seed only” detection option). Since we were not interested in repeat types and families, but only in repeats as potential drivers of genome rearrangements, we mapped the repeat pairs to the genome and calculated the percentage of the genome covered by repeats (Treangen *et al.* 2009) for all the species in our data sets. We calculated this repeat genome coverage separately for the repeats obtained by preset *P*-value threshold (*P* < 0.001), and for the repeats obtained by setting their lower length limit to 100 bp.

### Genome alignments

Pairs of genomes within data sets were aligned by the mummer program from the MUMmer package (Kurtz *et al.* 2004). Option “–mum” was used to detect maximal unique matching sequences (MUMs) between two genomes. MUMs correspond to regions homologous between the two genomes being aligned. Minimal length of MUMs was set to 20 bp for the P_arcticum data set, and to 25 bp for the D_radiodurans and E_faecium data sets (the minimum was raised to 25 bp to avoid noise in visualization). One of the genomes serves as a reference sequence against which the other genome is matched by the mummer program, in both forward and the reverse complement state. Coordinates of the matches were plotted as dot plots.

### Genomic indicators of selection

Prokaryotic genome organization is considered to be streamlined, exhibiting features that enable fast reproduction of cells (Rocha 2008; Koonin 2009). The prokaryotic genome organization is defined by multiple adaptive features that limit genome plasticity, *e.g.*, increased percentage of genes on the leading strand is expected to reduce clashes between DNA replication and transcription, and decreased distance of rRNA genes from the origin of replication is expected to increase the dosage of these highly expressed genes by early DNA replication (Rocha 2008). To quantify these replication-related indicators of selection for genome organization, positions of origins of replication were obtained from the DoriC database (Gao and Zhang 2007; Gao *et al.* 2013). The center of the origin area reported by DoriC was used as the origin position (listed in Table S2 in File S1), and the point half a genome away from the origin was used as the terminus position. Genomes for which the origin position was unknown were excluded from analyses of origin-dependent indicators of selection (Table S2 in File S1: 0–35% of the data sets, and the whole C_thermalis data set because it lacked the oriC position for one of the two radiation-resistant genomes in the data set).

We calculated several indicators of selection for the structural organization of genomes: (i) percentage of genes on the leading strand, for which we determined the leading strand as the one that contained more genes; (ii) the average distance of rRNA genes from the origin of replication; (iii) number of rRNA genes; (iv) ΔGC skew which is calculated as (*G*_leading_ − *C*_leading_)/(*G*_leading_ + *C*_leading_) − (*G*_lagging_ − *C*_lagging_)/(*G*_lagging_ + *C*_lagging_), *C* and *G* being the counts of *C* and *G* nucleotides on the leading and lagging replication strands of the genome, with the leading strand being defined as the one with more *G*’s; and (v) the number and length of stretches of genes in the same orientation on the genome, the analysis of which is indicative of the operon structure of the genome—we report the ratio of single genes (not preceded or followed by the gene in the same orientation, and thus not belonging to an operon) and the total number of genes. We used one-tailed *t*-tests to determine whether selection is more relaxed in radiation-resistant than in nonresistant genomes.

### Data availability

All the GenBank species used for analysis, organized by the data sets to which they belong are listed in Table S1 in File S1. Also listed are genome stability indices for each species, calculated using the model in Equation 1 fitted to the pooled GOC_250_ *vs.* 16S rRNA distance points of all the data sets (“modelAll” shown in Figure 2A) or calculated using within-data set models (models shown in Figure 2C and in the inset of Figure 3). Also given are the positions of origins of replication from the DoriC database (Gao and Zhang 2007; Gao *et al.* 2013) used for calculations of genomic indicators of selection, D_10_ values found in the literature, and supplementary references. Table S3 in File S1 contains parameters *f*_i_ and *p* and their confidence intervals for the model in Equation 1 fitted to the GOC_250_ *vs.* rRNA data, separately for different data sets. Custom code used for the analyses can be found at https://github.com/jrepar/Rearrangements.

## Results

To study genome-rearrangement rates, we compare the loss of gene order conservation with time between each of the two species in a data set (time is estimated as 16S rRNA distance). To look at the gene order conservation between two species we first identify a common set of genes, *i.e.*, orthologs. From the different arrangements of orthologs, we estimate the genome-rearrangement distance between species; the relationship between rearrangement distance and evolutionary time allows us to compare the dynamics of genome rearrangements (i) between different prokaryotic clades, and (ii) within clades, between radiation-resistant and nonresistant prokaryotes.

### Information on rearrangement distance between genomes is preserved in the subsets of orthologs

**Figure 1:**
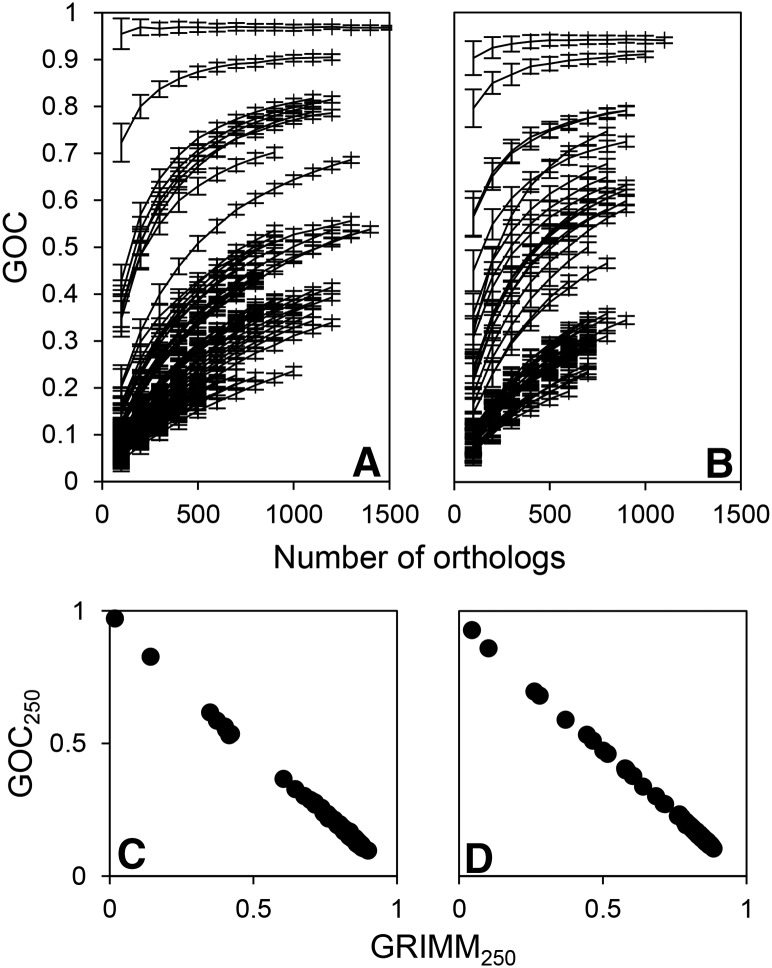
We used two different estimates of rearrangement distance between genomes: GOC (based on the portion of ortholog neighbors between which no breakpoint has been introduced) and GRIMM (based on the number of steps needed to transform gene order of one genome into another by inversions). A pair of genomes with an identical sequence of orthologs have GOC of 1 and GRIMM of 0. Shown is the dependence of GOC on the number of (randomly subsampled) orthologs for genome pairs in (A) D_radiodurans and (B) T_gammatolerans data sets. Each data point is based on 100 resamplings from the detected ortholog pool shared by two genomes. Error bars represent SD of GOC. Also shown is the relationship of the two estimates of rearrangement distance calculated from random ortholog subsets (GOC_250_ and GRIMM_250_, see main text) for (C) D_radiodurans and (D) T_gammatolerans data sets.

We identified orthologs between each genome pair within each data set. The number of orthologs shared between genome pairs varies considerably both between and within our data sets (Table 2). Minimum, maximum, and average number of orthologs shared between genome pairs within different data sets are listed in Table 2. Next, we checked whether the variation in the number of orthologs shared by different pairs of species might influence our estimates of rearrangement distance between genomes. We used two different estimates of rearrangement distance between genomes: GOC (based on the portion of orthologs contiguous in both genomes, *i.e.*, the portion of ortholog neighbors between which no breakpoint has been introduced) and GRIMM (based on the number of steps needed to transform the gene order of one genome into another by inversions, see *Methods*). By simulating random loss of orthologs, we observed that the rearrangement distance between two genomes, measured by GOC, depends on the total number of orthologs they share (Figure 1, A and B). Therefore, to make rearrangement distances comparable on a larger evolutionary scale, we calculated the GOC and GRIMM measures on randomly sampled ortholog subsets of equal size (*n* = 250) multiple times, and used the average values as estimates of rearrangement distance (see *Methods*). The thus obtained GOC_250_ and GRIMM_250_ measures correlate very well (Pearson’s *R* for all data sets >0.99, example data sets shown in Figure 1, C and D). Low SD of both GOC_250_ and GRIMM_250_, as well as the strong correlation between GOC_250_ and GOC_100_ measures (differing in the number of subsampled orthologs, *e.g.*, Pearson’s *R* = 0.97 for the D_radiodurans data set), show that the random sample of orthologs produces consistent estimates of rearrangement distance.

**Table 2:** To compare gene order between any two species, we identified their common set of genes, *i.e.*, orthologs

Data set	Maximum number of orthologs	Minimum number of orthologs	Average number of orthologs
D_radiodurans	2044	758	1235
E_faecium	4278	564	1123
P_arcticum	3111	597	1128
M_radiotolerans	5332	1039	1954
T_gammatolerans	1639	307	664
K_radiotolerans	3722	613	1355
C_thermalis	4307	1473	2403

Orthologs were identified for each species pair within each data set. The numbers of orthologs between species pairs vary; the table presents minimal, maximal, and average number of orthologs detected between a species pair for each data set.

We also analyzed whether the stringency of ortholog definition influences our measure of rearrangement distance. For the D_radiodurans data set, we examined how GOC_100_ depends on the sequence-identity threshold required to declare best bidirectional blast hits as orthologs (GOC_100_ was used to ensure that there will be enough orthologs for the comparisons at the higher identity threshold). Besides the 40% identity threshold used for all the data sets, for D_radiodurans we have also considered 20, 30, and 50% thresholds (number of orthologs for each threshold listed in Table S1 in File S1). The GOC_100_ calculated by using different thresholds correlates well between the same organism pairs within the D_radiodurans data set (Pearson’s *R* > 0.96 for comparisons between the default 40% threshold with other thresholds). Lowering the percentage identity threshold may introduce paralogs into the sets of presumably orthologous genes shared between genome pairs. However, this does not affect our rearrangement-distance measure, which is based on the ortholog resampling and distance averaging (see *Methods*). It follows from the above described results that the rearrangement distance can be accurately estimated from relatively small subsets of the genes shared between two genomes.

### Rates of genome rearrangements vary between phylogenetic clades

**Figure 2:**
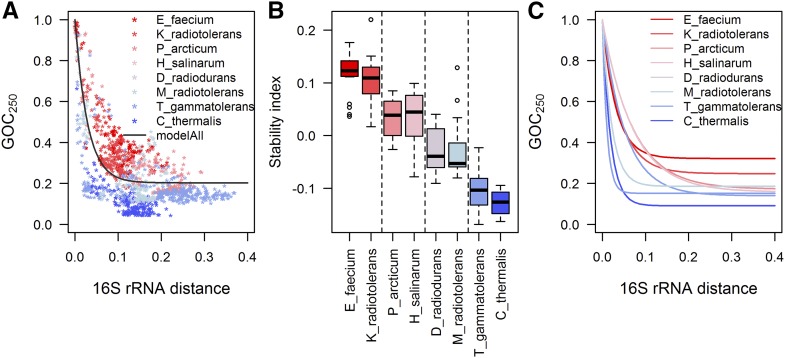
(A) Loss of gene order conservation (GOC_250_) with time (time is estimated through 16S rRNA distance, in nucleotide substitutions per site). Different data sets are shown in different colors and have different rates of GOC_250_ change, implying different rates of genome rearrangements. GOC_250_ quantifies rearrangements between pairs of genomes through the change of gene order; two genomes with an identical sequence of orthologs have GOC_250_ of 1. As the order of orthologs between two species diverges through time, GOC_250_ decreases toward 0, because it represents the gene order conservation, *i.e.*, the portion of ortholog neighbors between which no breakpoint has been introduced. (B) To further investigate differences in genome rearrangement rates between data sets, we calculated stability indices for each species. Each species participates in multiple GOC_250_-16S rRNA distance points. Genome-stability index for a species shows average deviation of these points from the typical group rearrangement dynamics, represented here by “modelAll” in (A). Therefore, stability indices represent deviation of observed GOC_250_ from the expected GOC_250_ at a given divergence time point (if there was no deviation, stability index would be 0, whereas the genome stability index of 0.1 means that 10% of the ortholog neighbors that would be expected to have a breakpoint between them are, instead, preserved). Genome stability indices in plot (B) are grouped by data set. The vertical lines in (B) show significantly different means of genome-stability indices as compared by Tukey–Kramer HSD test (at *α* = 0.05). (C) Independent model fits for different data sets further characterize the different dynamics of accumulation of genome rearrangements for each of the data sets. Parameters of all the fits of the model in Equation 1 to different (sub-) sets of data and their confidence intervals are given in Table S3 in File S1. For comparison of rearrangement dynamics, at each 16S rRNA distance between two species one can read out the GOC_250_ values to estimate the portion of still-contiguous orthologs. For example, at the 16S rRNA distance of 0.075, the typical GOC_250_ predicted by the model for slow rearranging E_faecium data set and fast rearranging C_thermalis data set is 0.383 and 0.108, respectively. Therefore, at this divergence time point the portion of nonrearranged ortholog neighbors is 3.5 times lower in a typical E_faecium genome than in a typical C_thermalis genome.

Our data sets form distinct groups when the dynamics of genome rearrangements are compared through the relationship of GOC_250_ and 16S rRNA distance (Figure 2A). Since our data sets represent different phylogenetic clades, the grouping of their GOC_250_-16S rRNA distance points (Figure 2A) emphasizes internal clade consistency, *i.e.*, internal similarity in rates of genome rearrangements. This is an expected result from the viewpoint of phylogenetic inertia, and is in agreement with previous observations (*e.g.*, Rocha 2006).

We quantified the differences in genome stability between different clades by calculating genome stability indices for each species. Each species participates in multiple GOC_250_-16S rRNA distance points (because points represent genome pairs, *e.g.*, Figure 2A). The genome-stability index for a species shows average deviation of these points from the typical group rearrangement dynamics. Here, to describe typical group rearrangement dynamics of all the clades jointly, we use the model fitted to their pooled GOC_250_-16S rRNA distance data (the model used is shown in Figure 2A as “modelAll,” and its parameter values can be found in Table S3 in File S1). Figure 2B compares genome stability indices for species grouped by clades. The Tukey–Kramer honest significant difference (HSD) test showed significant differences between mean stability indices of some data sets (Figure 2B).

Interestingly, species in the P_arcticum and M_radiotolerans data sets show different mean stability indices, *i.e.*, different rates of genome rearrangement, demonstrating that subclades from the same bacterial phylum can vary in genome stability (Figure 2B). The focal organisms around which these data sets were assembled, the *P. arcticum* and *M. radiotolerans*, belong to γ- and α-proteobacteria, respectively. As can be seen in Figure 2B, γ-proteobacteria show higher genome stability than α-proteobacteria, a result that is in agreement with previous observations (Rocha 2006).

The archaeal T_gammatolerans data set shows very low genome-stability indices, as low as the least stable bacterial data set (C_thermalis, Figure 2). To obtain more information on archaeal rates of genome rearrangements and genome stability, we included an additional data set—the H_salinarum data set—in the comparison of different clades (Figure 2; list of species in Table S2 in File S1). Both archaeal data sets belong to the phylum Euryarchaeota, but are built around species belonging to different classes: *H. salinarum* belongs to Halobacteria, while *T. gammatolerans* belongs to Thermococci. Figure 2 shows that the H_salinarum data set has higher genome-stability indices and lower rates of genome rearrangements when compared to the T_gammatolerans data set. Figure 2 demonstrates that genome-rearrangement rates in different archaeal clades can be more similar to the genome-rearrangement rates in some bacterial clades than to each other. This was confirmed by the statistical comparison of their genome-stability indices (Tukey–Kramer HSD test, Figure 2B).

### Higher genome-rearrangement rates in radiation-resistant species

We divided the species within each data set into two groups—based on whether they are radiation-resistant or not—and compared their (i) genome-rearrangement rates, and (ii) genome-stability indices. Since the GOC_250_ *vs.* 16S rRNA distance points show strong clade-dependent patterns (Figure 2A), consistent with the influence of phylogenetic inertia, we modeled each data set separately.

**Figure 3:**
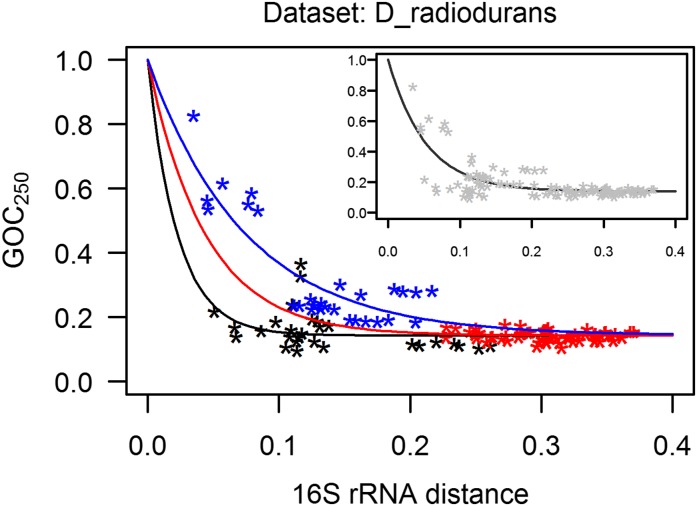
Loss of gene order conservation (GOC_250_) with time, for D_radiodurans data set (time is estimated through 16S rRNA distance, expressed in nucleotide substitutions per site). Each point represents a genome pair; the three categories of points (shown in different colors) were defined by radiation resistance (R) or nonresistance (N) of species in the genome pair. Model describing the R-R category of points is shown in black, the R-N category in red, and the N-N category in blue. The steeper the decline of the model fits, the higher the rate of rearrangements. At each 16S rRNA distance between two species one can read out the GOC_250_ values to estimate the portion of still-contiguous orthologs. For example, at the 16S rRNA distance of 0.075, the typical GOC_250_ predicted by the models for R-R and N-N category of points is 0.174 and 0.457, respectively. Therefore, at this divergence time point, the portion of nonrearranged ortholog neighbors is 2.6 times lower in a typical radiation-resistant species than in a typical nonresistant species. (Inset) Rearrangement dynamics of the whole D_radiodurans group is described by the model fit to all the GOC_250_ 16S rRNA distance points in the data set; this model was used for calculation of genome-stability indices for the statistical comparison of radiation-resistant and nonresistant species and as a reference for statistical comparison of residuals of different categories of points, as described in main text.

The D_radiodurans data set shows higher rates of genome rearrangements for radiation-resistant species when compared to nonresistant species (Figure 3). The difference in rearrangement rates is visible in the different rates of decline of the model fits for the different categories of GOC_250_ *vs.* 16S rRNA distance points—the steeper the decline of the model fit, the faster the rate of genome rearrangements (Figure 3). The 95% confidence intervals for parameters of the models, including the parameter *p* which estimates the rate of decline, are given in Figure S1 and Table S3 in File S1 and they show a significant difference in the rate of decline for different categories of points in the D_radiodurans data set.

Genome-stability indices for the species in the D_radiodurans data set were calculated with reference to the model shown in the inset of Figure 3: the genome-stability indices (Table S2 in File S1) are significantly lower for the radiation-resistant species when compared to nonresistant species (*P*-value = 7.77e−05, exact two-sample Fisher–Pitman permutation test, two-tailed).

Each GOC_250_-16S rRNA distance point within a data set represents two species; the points can be categorized with respect to the radiation resistance of the two species for which the point was calculated. Such categories can then be compared directly instead of averaging the points into an overall genome-stability index for a species. We therefore compared the residuals calculated for the pooled R-R and R-N category of points *vs.* the residuals for the N-N category of points (named for the radiation resistance of the species in the pair: R denotes resistant, and N denotes nonresistant species). The points that include a radiation-resistant species are significantly shifted toward more genome rearrangements (*P*-value < 2.2e−16, two-sample Fisher-Pitman permutation test, two-tailed).

**Figure 4:**
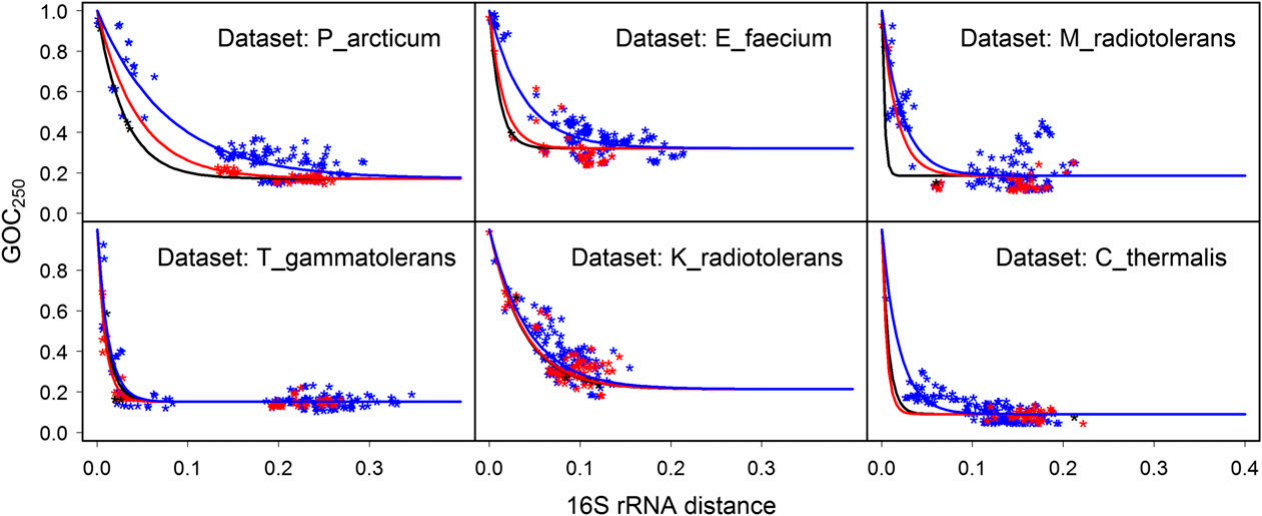
Loss of gene order conservation (GOC_250_) with time, for six data sets noted in the top right corner of each plot (time is estimated through 16S rRNA distance, expressed in nucleotide substitutions per site). Each point represents a genome pair; the three categories of points, shown in different colors, were defined by radiation resistance (R) or nonresistance (N) of species in the genome pair. Model describing the R-R category of points is shown in black, the R-N category in red, and N-N category in blue. Faster decline of the model fits signifies higher rate of genome rearrangement.

We checked whether the observation of elevated rate of genome rearrangements in radiation-resistant species of the D_radiodurans data set can be generalized to other prokaryotic clades. The fits of the model to three categories of points (N-N, N-R, and R-R) for six additional data sets (Figure 4) show a consistently higher rate of genome rearrangements (faster decline of the model fits) for the categories that include radiation-resistant species, though the difference between rates is not strong for some data sets. The 95% confidence intervals for parameter *p*, which estimates the rate of decline of model fits, are completely nonoverlapping (*i.e.*, parameter *p* is significantly different) for the three categories of points for the data sets of P_arcticum, E_faecium, and M_radiotolerans, given a fixed parameter *f_i_* (Table S3 in File S1).

We tested the difference between genome-stability indices (Table S2 in File S1) of radiation-resistant and nonresistant species and obtained *P*-values of 0.005, 0.02, 0.17, 0.28, 0.41, and 0.83 for the P_arcticum, E_faecium, M_radiotolerans, T_gammatolerans, K_radiotolerans, and C_thermalis data sets, respectively (exact two-sample Fisher–Pitman permutation test, two-tailed). Even though *Halobacterium salinarum* is radiation resistant (Kotteman *et al.* 2005), we did not include the H_salinarum data set in these analyses due to the scarcity of radiation-resistant data points for this set (only one known resistant species). We also compared the residuals calculated for the category of points containing radiation-resistant species (R-R and R-N points) and the residuals for the N-N category of points by the two-sample Fisher–Pitman permutation test, two-tailed, in the context of the models for whole data sets (models shown in Figure 2C). Resulting *P*-values were <2.2e−16 for the data sets of P_arcticum and E_faecium, and 0.0018, 0.034, 0.044, and 0.62 for the data sets M_radiotolerans, T_gammatolerans, K_radiotolerans, and C_thermalis, respectively. These results confirm the finding of elevated rates of genome rearrangements in radiation-resistant species in additional data sets (most strongly for P_arcticum and E_faecium), phylogenetically independent from the D_radiodurans data set, and from each other.

Interestingly, despite the phylogenetic inertia, when we compared stability indices calculated with reference to the modelAll in Figure 2A (model fitted to the data points of all data sets pooled together) for all radiation-resistant and nonresistant genomes, possible global tendency toward lower genome stability for radiation-resistant species emerged (*P*-value = 0.037, exact two-sample Fisher–Pitman permutation test, two-tailed, when using all the nonresistant genomes; and *P*-value = 0.0004 when using only the confirmed nonresistant genomes. See *Methods* and Table S2 in File S1 for classification of genomes and stability-index values).

### Causes of elevated rearrangement rate in radiation-resistant species

#### Elevated rate of genome rearrangements in radiation-resistant species is not due to the higher repeat content:

For each species, we measured the percentage of the genome covered by two types of repeats: (i) exact repeats of length roughly corresponding to the length of MEPS, and (ii) exact repeats of a minimal length of 100 bp. We detected no significant difference in the percentage of genome covered by repeats between radiation-resistant and nonresistant species in any data set for either type (*P* > 0.05 for all comparisons, unequal variance *t*-tests, two-tailed). Our analysis detected no avoidance of repetitive sequences in the genomes of radiation-resistant species, even though such avoidance would be expected to help in accurate genome reassembly.

#### Fast gene order loss in radiation-resistant species does not result from relaxed selection against genome rearrangements:

Multiple selective constraints oppose genome rearrangements in prokaryotic genomes (Rocha 2008). We examined several indicators of such selective constraints and found no consistent pattern in terms of differences between radiation-resistant and nonresistant species within data sets: *P*-values were above or close to 0.05 for most measures for most data sets (unequal variance *t*-tests, one-tailed).

In particular, the genomic indicators of selection against genome rearrangements that we examined are: (i) ratio of genes in the genome that are not followed or preceded by the gene in the same orientation, (ii) average distance of the rRNA genes from the origin of replication [both (i) and (ii) are expected to be lower in the more structured genomes], (iii) percentage of genes on the leading strand, (iv) number of rRNA genes, and (v) ΔGC skew (the three latter ones are expected to be higher in the more structured genomes). When comparing radiation-resistant and nonresistant genomes, of 35 comparisons (five per data set), we found no significant differences in most measures for most data sets (*P* > 0.05, unequal variance *t*-tests, one-tailed). Exceptions, per data set, were the ΔGC skew and number of single genes for the D_radiodurans data set (*P* = 0.019 and 0.0033), ΔGC skew and number of genes on the leading strand for the M_radiotolerans data set (*P* = 0.0018 and 0.007, respectively), rRNA distance from oriC for the E_faecium data set (*P* = 0.009), and lower percentage of genes on the leading strand in the radiation-resistant genomes of the K_radiotolerans data set (*P* = 0.024). These *P*-values are not significant if Bonferroni correction is applied. No differences for either measure were detected for the P_arcticum and T_gammatolerans data set. Overall, there is no support for the relaxed selection on gene order in the radiation-resistant species.

#### The dominant prokaryotic mechanism of genome rearrangements is also shaping the genomes of radiation-resistant species:

An X shape is sometimes visible in pairwise genome alignments of both radiation-resistant and nonresistant species in our data sets (Figure 6), indicating that inversions around the origin of replication play an important role in genome rearrangements in both groups of genomes. This is further corroborated by the high correlation of rearrangement distances based on breakpoints, and those based on transforming gene order of one genome into another by inversions (Pearson’s *R* of GOC_250_ and GRIMM_250_ for all the data sets is >0.99; shown for D_radiodurans and T_gammatolerans data sets in Figure 1, C and D); inversions explain change of gene order well. High correlation between the two types of measures has also been previously observed for γ-proteobacteria (Belda *et al.* 2005).

## Discussion

We found historical genome-rearrangement rates to be higher in radiation-resistant species than in phylogenetically close, nonresistant species.

We expect the bias toward higher rearrangement rates in radiation-resistant species to be affected by the extreme environments that they are adapted to survive in. These environments (ionizing radiation, desiccation) cause a high incidence of DSBs, which can cause genome rearrangements through ectopic recombination (Kowalczykowski *et al.* 1994; Argueso *et al.* 2008; Repar *et al.* 2010). It is plausible, therefore, that the high incidence of DSBs, a consequence of the environmental conditions, is a driver of the higher rate of genome rearrangements in the radiation-resistant species.

The higher rearrangement rates might also be helping organisms adapt to stress by introducing potentially beneficial new mutations through genome reshuffling, but the lack of difference in selection for genome organization between radiation-resistant and nonresistant species does not suggest that increase in rearrangement rates is an important contributor to surviving stress.

In contrast to the majority of phylogenetic clades tested, several clades did not show significant negative correlation between radiation resistance and genome stability. There are several possible reasons for the lack of correlation. First, availability of completely sequenced species can introduce bias, as can the rate of genome rearrangements within a clade. For example, in the fast rearranging data set of C_thermalis, the pairwise comparisons that include the two radiation-resistant bacteria fall in the rearrangement-saturation part of the GOC_250_ *vs.* 16S rRNA distance curve, where we would expect values for both resistant and nonresistant bacteria to behave similarly. However, fits to the different categories of points (R-R, R-N, and N-N) still indicate that there might be differences in rates of genome rearrangements of radiation-resistant and nonresistant species in this data set (Figure 4). Second, the conservative approach in classification (see *Methods*) and the small number of radiation-resistant species within each data set may have reduced the statistical power of analysis. Third, level of radiation resistance varies even among the species classified as radiation resistant (Table S2 in File S1). The variability may be reflected in traits that correlate with radiation resistance (*e.g.*, rearrangement rates), thus possibly further reducing the statistical power of analysis.

**Figure 5:**
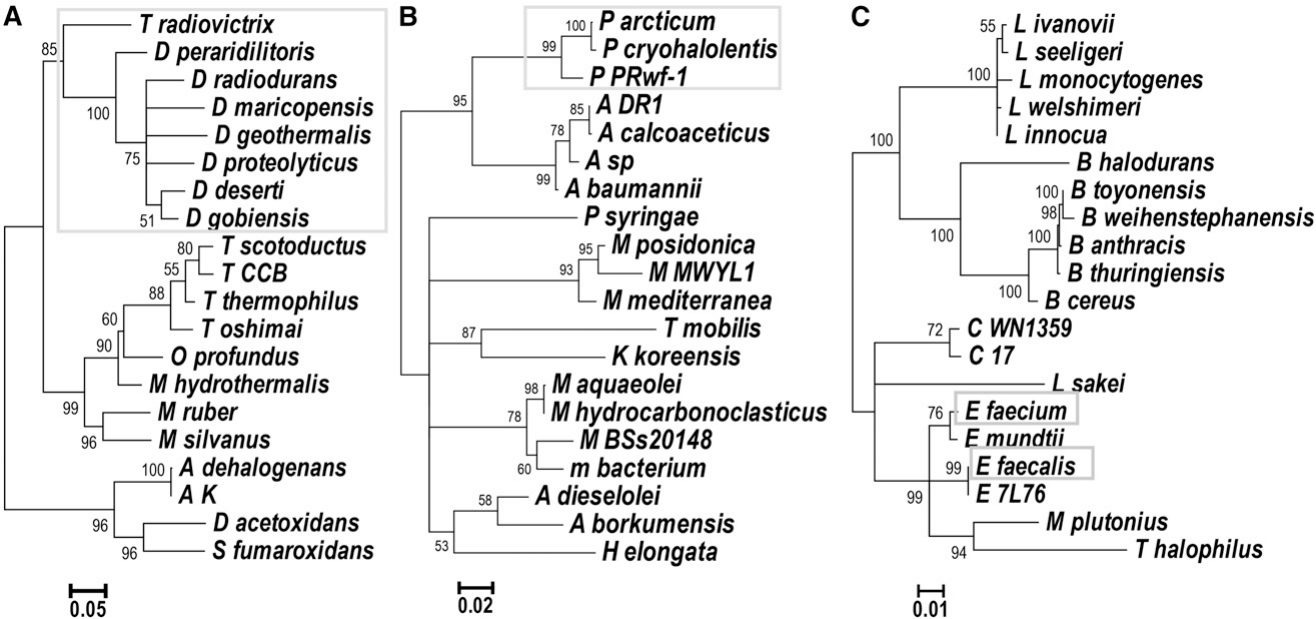
Maximum likelihood trees obtained from 16S rRNA alignments for data sets (A) D_radiodurans, (B) P_arcticum, and (C) E_faecium. (A) D_radiodurans tree contains four additional species from a different phylum (*Anaeromyxobacter dehalogenans*, *A. sp. K*, *Desulfobacca acetoxidans*, and *Syntrophobacter fumaroxidans*). Branches with radiation-resistant species are marked with gray boxes. Full species names are given in Table S2 in File S1.

The 16S rRNA substitution rates were used throughout this analysis as the measure of time available for the accumulation of rearrangements. Variability of 16S rRNA substitution rates would influence our conclusions: the rearrangement rate might appear faster in radiation-resistant species if they accumulated a lower number of 16S rRNA substitutions in the available time. Several lines of evidence argue for higher rearrangement rates. First, the background mutation rate of a representative radiation-resistant species, bacterium *D. radiodurans*, in the absence of radiation is similar to that of the nonresistant *E. coli* (Long *et al.* 2015). This suggests that the DNA repair systems that help *D. radiodurans* survive the mutagenic effects of UV (Sweet and Moseley 1974), do not decrease its background accumulation of DNA substitutions. Second, the maximum likelihood phylogenetic trees we obtained from 16S rRNA using TreePuzzle show similar lengths of branches leading to radiation-resistant species and branches leading to phylogenetically related, nonresistant species (Figure 5). Therefore, we conclude that the differences in genomic-stability indices of radiation-resistant species stem from higher rearrangement rates and not from variable 16S rRNA rates.

In general, rate of gene order loss in prokaryotic genomes correlates with their repeat content (Zivanovic *et al.* 2002; Rocha 2003). Repetitive sequences are relevant for genome rearrangements in the context of repair of DSBs; recombination repair using homologous sequences, although generally accurate, may be misled by ectopic repetitive sequences (Shen and Huang 1986; Kowalczykowski *et al.* 1994). Avoidance of repetitive sequences would plausibly help accurate genome reassembly in the DSB-causing environments. However, we detect no significant difference in repeat content between radiation-resistant and nonresistant species. This is in agreement with the previous finding that the abundance of both short and long repetitive sequences in *D. radiodurans* is comparable to *E. coli* (Makarova *et al.* 1999, 2001). This also means that repeat content does not cause elevated rates of genome rearrangements in radiation-resistant species.

**Figure 6:**
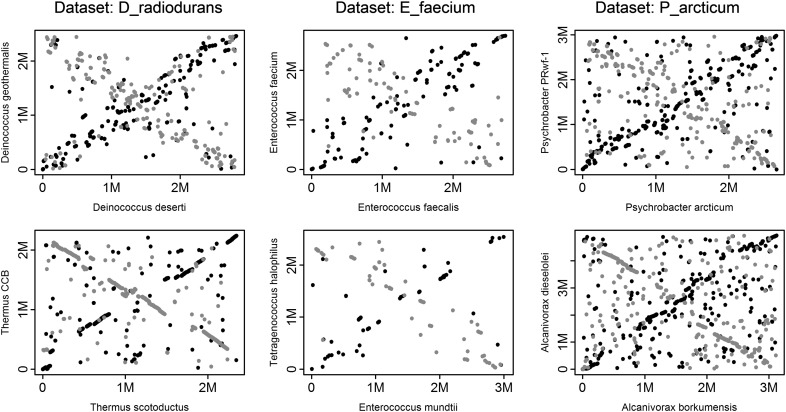
Dot plots show positions of homologous sequences between pairs of genomes (in base pairs, where *M* denotes 1 million). Homologous sequences between two genomes are represented by MUMs (see *Methods*). Shown are positions of MUMs identified between the forward conformation of the genome on the *x*-axis and the forward conformation of the genome on the *y*-axis (black •), or the reverse conformation of the genome on the *y*-axis (gray •). Only the largest genome element is shown for the multi-element genomes (see *Methods*). Each column represents one data set; for each data set shown, the first plot is a MUM alignment of two radiation-resistant genomes, and the second plot a MUM alignment of two nonresistant genomes.

Footprints of inversions symmetrical around the origin of replication are visible in the rearrangement history of some radiation-resistant and nonresistant genomes (Figure 6). It has been suggested that symmetrical inversions arise during genome replication and are associated with repair of collapsed replication forks (Tillier and Collins 2000). In general, collapse of replication forks might be an important contributor to the genome rearrangements in radiation-resistant (similar to nonresistant) species (Michel *et al.* 2001). For example, *D. radiodurans* does not lose viability and continues to multiply under high doses of gamma radiation (60 Gy/h; Lange *et al.* 1998); since the genome of *D. radiodurans* is not specially protected from the damaging effects of radiation (Daly 2012), damage to genomic DNA, *e.g.*, DSBs or single-stranded breaks, can cause collapse of replication forks (Kowalczykowski 2000).

Interestingly, the finding of conserved positioning of essential genes relative to the origin of replication in very rearranged Thermococcales chromosomes (Cossu *et al.* 2015) is consistent with symmetrical inversions as an important source of gene order change in these species; symmetrical inversions are not expected to change the distance of genes to the origin of replication (Eisen *et al.* 2000; Mackiewicz *et al.* 2001). In general, symmetrical inversions are not expected to perturb important, replication-related structural characteristics of genomes (Eisen *et al.* 2000; Mackiewicz *et al.* 2001) and would, therefore, be in agreement with the finding that the selection against genome rearrangements is not relaxed in radiation-resistant species.

The extent to which genetic drift influences populations of radiation-resistant microbes is unclear. Low environmental concentration (Slade and Radman 2011) and population bottlenecks possibly caused by desiccation periods would be expected to increase the influence of genetic drift. Such conditions would relax selection, including selection against genome rearrangements. Since the organized genome structure of prokaryotes results from selection (Rocha 2008, Koonin 2009), the relaxed selection would result in less structured genomes. However, we do not observe evidence of relaxed selection on gene order in radiation-resistant species when compared to phylogenetically close, nonresistant species.

Several lines of evidence point toward the significance of adapted DNA repair in ionizing radiation survival, evidence such as the accurate and efficient genome reassembly after hundreds of DSBs in *D. radiodurans* and other radiation-resistant prokaryotes (*e.g.*, Zahradka *et al.* 2006; Repar *et al.* 2010; Confalonieri and Sommer 2011); a specific DSB repair mechanism in *D. radiodurans* (Zahradka *et al.* 2006; Slade *et al.* 2009); positive selection acting on DNA repair genes in radiation-resistant bacteria, in contrast to nonresistant bacteria (Sghaier *et al.* 2008); and optimization of DNA repair functions in the ionizing-radiation-resistant *E. coli* obtained in the laboratory by directed evolution (Byrne *et al.* 2014). The notion of enhanced DSB repair operating in radiation-resistant species is seemingly in disagreement with the high rates of genome rearrangement we observed in their genomes. However, high rates of rearrangement do not preclude the possibility that a DNA repair system has evolved to be more accurate in the radiation-resistant species; the assault on DNA might just be too strong to be compensated for. Regardless, our conclusions are restricted by the fact that our data does not measure specific (and opposing) contributions of DNA damage and DNA repair but instead looks at the sum of their effects.

In conclusion, ionizing-radiation-resistant species show higher than expected evolutionary rates of genome rearrangements as well as decreased genome stability; the opposing effects of DNA damage and DNA repair result in faster net gain of genome rearrangements in radiation-resistant species when compared to phylogenetically close, nonresistant species.

## Supplementary Material

Supplemental material is available online at www.genetics.org/lookup/suppl/doi:10.1534/genetics.116.196154/-/DC1.
