# Supplementary material for: Elevated Rate of Genome Rearrangements in Radiation-Resistant Bacteria
Source: Genetics. 2017 Feb 10;205(4):1677–89. doi: 10.1534/genetics.116.196154 (PMC5378121; doi:10.1534/genetics.116.196154)
Supplement: Supplementary file 1 [file 1677file001.docx]

**SUPPLEMENTARY DATA**

**Table S1** Dependence of the number of orthologs detected between species pairs on the stringency of ortholog definition. Orthologs were detected as best bidirectional blast hits with additional conditions, including %identity. Here, we show statistics of orthologs detected at different %identity tresholds for the D_radiodurans dataset. 40% identity threshold is used for defining orthologs throughout the paper.

| % identity used for ortholog definition | Max number of orthologs | Min number of orthologs | Average number of orthologs |
| --- | --- | --- | --- |
| 20% | 2208 | 1029 | 1467 |
| 30% | 2157 | 976 | 1419 |
| 40% | 2044 | 758 | 1235 |
| 50% | 1947 | 442 | 959 |

**Table S2** List of all the species used for analysis, organized by the datasets they belong to. Also listed are genome stability indices for each species, calculated using model (1) fitted to the pooled GOC_250_ vs. phylogenetic distance points of all the datasets (“modelAll” shown in Figure 2a) or calculated using within-dataset models (models shown in Figure 2c and in the inset of Figure 3). Also given are the positions of origins of replication from DoriC (Gao and Zhang 2007; Gao *et al.* 2013) used for calculations of genomic indicators of selection, and D_10_ values found in the literature (the D_10_ value was estimated from radiation survival curve if not explicitly stated in the reference, not available for all the species of known radiation-resistance because even though all have been found to survive large doses of ionizing radiation, for some of them no survival curves were measured). Names of known radiation-resistant species are marked with an asterisk (*), of known radiation-sensitive species with an ampersand (&); the rest are of unknown radiation resistance (conservatively classified for the analysis as non-resistant, see Methods).

| Dataset name | GenBank species name (radiation resistant species are shown in red) | Stability index (calculated to the within-dataset model) | Stability index (calculated to the model describing all the datasets together) | Position of origin of replication (from DoriC) | D_10_ value for survival of gamma radiation and references |
| --- | --- | --- | --- | --- | --- |
| C_thermalis | Anabaena_90_uid30803 | 0.0327 | -0.0950 |  |  |
|  | Anabaena_cylindrica_PCC_7122_uid43355 | 0.0196 | -0.1185 |  |  |
|  | Arthrospira_platensis_NIES_39_uid42161 (*) | -0.0127 | -0.1274 |  | >1200 for Spirulina platensis (Zhiping *et al.* 1998) |
|  | Anabaena_variabilis_ATCC_29413_uid10642 | -0.0175 | -0.1528 |  |  |
|  | Chamaesiphon_PCC_6605_uid158825 | -0.0341 | -0.1500 |  |  |
|  | Cyanothece_PCC_7425_uid28337 | -0.0336 | -0.1509 |  |  |
|  | Calothrix_PCC_7507_uid158683 | -0.0014 | -0.1446 |  |  |
|  | Crinalium_epipsammum_PCC_9333_uid158835 | 0.0217 | -0.0944 |  |  |
|  | Cylindrospermum_stagnale_PCC_7417_uid158809 | 0.0125 | -0.1294 |  |  |
|  | Chroococcidiopsis_thermalis_PCC_7203_uid38119 (*) | 0.0042 | -0.1094 |  | 3750-5000 for Chroococcidiopsis spp. (Billi *et al.* 2000) |
|  | Geitlerinema_PCC_7407_uid158833 | 0.0133 | -0.1047 |  |  |
|  | Gloeocapsa_PCC_7428_uid158831 | 0.0261 | -0.0951 |  |  |
|  | Microcoleus_PCC_7113_uid158839 | -0.0008 | -0.1217 |  |  |
|  | Nostoc_PCC_7524_uid158707 | 0.0025 | -0.1347 |  |  |
|  | _Nostoc_azollae__0708_uid30807 | 0.0355 | -0.1046 |  |  |
|  | Nostoc_punctiforme_PCC_73102_uid216 | 0.0219 | -0.1095 |  |  |
|  | Nostoc_sp_uid244 | -0.0265 | -0.1627 |  |  |
|  | Oscillatoria_PCC_7112_uid158711 | -0.0093 | -0.1250 |  |  |
|  | Synechocystis_PCC6803_uid60 | -0.0436 | -0.1584 |  |  |
|  | Stanieria_cyanosphaera_PCC_7437_uid158877 | -0.0288 | -0.1456 |  |  |
| E_faecium | Bacillus_anthracis_H9401_uid49361 | 0.0205 | 0.1278 | 1836 |  |
|  | Bacillus_cereus_cytotoxis_NVH_391-98_uid13624 (&) | 0.0572 | 0.1766 | 1952 | 173-400 for B. cereus (Grant and Patterson 1992, Kotiranta *et al.* 1999) |
|  | Bacillus_halodurans_uid235 | 0.0471 | 0.1743 | 285 |  |
|  | Bacillus_thuringiensis_serovar_finitimus_YBT_020_uid60447 | 0.0181 | 0.1261 | 1837 |  |
|  | Bacillus_toyonensis_BCT_7112_uid225857 | 0.0241 | 0.1310 |  |  |
|  | Bacillus_weihenstephanensis_KBAB4_uid13623 | 0.0324 | 0.1427 | 1537 |  |
|  | Carnobacterium_17_4_uid60607 | -0.0043 | 0.1202 | 1530 |  |
|  | Carnobacterium_WN1359_uid222287 | 0.0114 | 0.1359 |  |  |
|  | Enterococcus_7L76_uid39181 | -0.0828 | 0.0362 |  |  |
|  | Enterococcus_faecalis_OG1RF_uid20843 (*) | -0.0791 | 0.0400 | 1503 | 1566 for Streptococcus faecalis (Garcia *et al.* 1987) |
|  | Enterococcus_faecium_DO_uid30627 (*) | -0.0623 | 0.0596 | 1504 | 900-2000 (Daly *et al.* 2004, Annelis *et al.* 1973) |
|  | Enterococcus_mundtii_QU_25_uid192584 | -0.0691 | 0.0534 |  |  |
|  | Listeria_innocua_uid86 (&) | 0.0058 | 0.1114 |  | 660-720 for Listeria innocua irradiated in gelatin (Rodriguez *et al.* 2006) |
|  | Listeria_ivanovii_uid13441 | 0.0064 | 0.1134 | 1770 |  |
|  | Listeria_monocytogenes_N53_1_uid177090 (&) | 0.0319 | 0.1412 |  | 160-650 (Patterson 1989, Grant and Patterson 1992, Saroj *et al.* 2006, Brandāo Areal *et al.* 1993) |
|  | Lactobacillus_sakei_23K_uid13435 | 0.0379 | 0.1611 | 1645 |  |
|  | Listeria_seeligeri_serovar_1_2b_SLCC3954_uid41123 | 0.0117 | 0.1191 | 1771 |  |
|  | Listeria_welshimeri_serovar_6b_SLCC5334_uid13443 | 0.0116 | 0.1187 | 1770 |  |
|  | Melissococcus_plutonius_ATCC_35311_uid61383 | 0.0373 | 0.1633 |  |  |
|  | Tetragenococcus_halophilus_uid41613 | -0.0082 | 0.1151 | 1414 |  |
| D_radiodurans | Deinococcus_deserti_VCD115_uid16691 (*) | -0.0389 | -0.0512 | 1617 | 7500 (Dulermo *et al.* 2009) |
|  | Deinococcus_geothermalis_DSM_11300_uid13423 (*) | -0.0051 | -0.0366 | 1829 | 5100-16000 (Ferreira *et al.* 1997, Daly *et al.* 2004, Makarova *et al.* 2007) |
|  | Deinococcus_gobiensis_I_0_uid46605 (*) | -0.0625 | -0.0715 | 1572 | 12700 (Yuan *et al.* 2009) |
|  | Deinococcus_maricopensis_DSM_21211_uid43461 (*) | -0.0225 | -0.0521 | 1759 |  |
|  | Deinococcus_peraridilitoris_DSM_19664_uid61295 (*) | -0.0123 | -0.0421 | 1940539 |  |
|  | Deinococcus_proteolyticus_MRP_uid41911 (*) | -0.0481 | -0.0703 | 1968 | 10300 (Shashidhar *et al.* 2010) |
|  | Deinococcus_radiodurans_R1_uid65 (*) | -0.0743 | -0.0907 | 1543 | 5500-16000 (Shashidhar *et al.* 2010, Battista 1997, Daly *et al.* 2004, Makarova *et al.* 2007) |
|  | Marinithermus_hydrothermalis_DSM_14884_uid50827 | 0.0262 | 0.0024 | 1526 |  |
|  | Meiothermus_ruber_DSM_1279_uid196343 | -0.0020 | -0.0457 |  |  |
|  | Meiothermus_silvanus_DSM_9946_uid29551 | 0.0431 | -0.0027 | 1590 |  |
|  | Oceanithermus_profundus_DSM_14977_uid40223 | 0.0326 | 0.0026 | 1518 |  |
|  | Thermus_CCB_US3_UF1_uid76491 | 0.0509 | 0.0403 | 1537 |  |
|  | Thermus_oshimai_JL_2_uid63181 | 0.0553 | 0.0382 | 1501 |  |
|  | Truepera_radiovictrix_DSM_17093_uid38371 (*) | -0.0145 | -0.0689 | 1665 | >5000 (Albuquerque *et al.* 2005) |
|  | Thermus_scotoductus_SA_01_uid46293 | 0.0510 | 0.0386 | 1514 |  |
|  | Thermus_thermophilus_HB8_uid13202 (&) | 0.0293 | 0.0231 | 1849646 |  |
| K_radiotolerans | Arthrobacter_FB24_uid12640 (*) | -0.0271 | 0.0842 | 1697 |  |
|  | Arthrobacter_aurescens_TC1_uid12512 (*) | -0.0422 | 0.0664 | 1707 |  |
|  | Arthrobacter_chlorophenolicus_A6_uid20011 | -0.0553 | 0.0565 | 1862 |  |
|  | Arthrobacter_nitroguajacolicus_Rue61a_uid78011 | -0.0522 | 0.0578 | 1807 |  |
|  | Arthrobacter_phenanthrenivorans_Sphe3_uid20087 | -0.0357 | 0.0741 | 2083 |  |
|  | Beutenbergia_cavernae_DSM_12333_uid20827 | 0.0001 | 0.1139 | 2061 |  |
|  | Blastococcus_saxobsidens_uid82915 (&) | 0.0137 | 0.1047 | 4875227 | 900 (Gtari *et al.* 2012) |
|  | Cellulomonas_fimi_ATCC_484_uid33691 | 0.0148 | 0.1233 | 1986 |  |
|  | Cellulomonas_flavigena_DSM_20109_uid19707 | 0.0143 | 0.1227 | 2049 |  |
|  | Cellvibrio_gilvus_ATCC_13127_uid33853 | 0.0163 | 0.1356 | 3492158 |  |
|  | Geodermatophilus_obscurus_DSM_43160_uid29547 (*) | 0.0015 | 0.0869 | 2427 | 9000 (Gtari *et al.* 2012) |
|  | Intrasporangium_calvum_DSM_43043_uid43527 | -0.0305 | 0.0913 | 2250 |  |
|  | Isoptericola_variabilis_225_uid49657 | 0.0015 | 0.1204 | 1906 |  |
|  | Kineococcus_radiotolerans_SRS30216_uid10689 (*) | 0.0016 | 0.0949 | 2027 | 7000 (Bagwell *et al.* 2008) |
|  | Kocuria_rhizophila_DC2201_uid27833 | 0.0378 | 0.1507 | 1999 |  |
|  | Kytococcus_sedentarius_DSM_20547_uid21067 | 0.0268 | 0.1277 | 2023 |  |
|  | Nakamurella_multipartita_DSM_44233_uid29537 | -0.0689 | 0.0166 | 2142 |  |
|  | Rothia_dentocariosa_ATCC_17931_uid48447 | 0.1164 | 0.2198 | 955933 |  |
|  | Sanguibacter_keddieii_DSM_10542_uid19711 | 0.0301 | 0.1432 | 1973 |  |
|  | Xylanimonas_cellulosilytica_DSM_15894_uid19715 | 0.0204 | 0.1318 | 1833 |  |
| P_arcticum | Acinetobacter_DR1_uid46105 | -0.0398 | -0.0017 | 4152526 |  |
|  | Acinetobacter_baumannii_BJAB0715_uid74423 (&) | -0.0300 | 0.0202 |  | 150-311 for Acinetobacter baumanii (Saha et Chopade 2009) |
|  | Alcanivorax_borkumensis_SK2_uid13005 | 0.0488 | 0.0799 | 3119889 |  |
|  | Acinetobacter_calcoaceticus_PHEA_2_uid51267 (&) | -0.0364 | 0.0016 | 2627250 | 150-170 (Saha et Chopade 2009, Nishimura *et al.* 1994) |
|  | Alcanivorax_dieselolei_B5_uid60443 | 0.0097 | 0.0560 | 4927892 |  |
|  | Acinetobacter_sp_ADP1_uid12352 | -0.0562 | -0.0162 | 3598489 |  |
|  | Halomonas_elongata_DSM_2581_uid49333 | 0.0104 | 0.0329 | 4061266 |  |
|  | Kangiella_koreensis_DSM_16069_uid29443 | 0.0674 | 0.0747 | 2852005 |  |
|  | Marinobacter_BSs20148_uid170720 | 0.0233 | 0.0833 | 4063630 |  |
|  | Marinomonas_MWYL1_uid17445 | 0.0045 | 0.0377 |  |  |
|  | Marinobacter_aquaeolei_VT8_uid13239 | 0.0118 | 0.0645 | 144 |  |
|  | Marinobacter_hydrocarbonoclasticus_uid91119 | 0.0141 | 0.0659 | 109 |  |
|  | Marinomonas_mediterranea_MMB_1_uid51765 | -0.0002 | 0.0396 | 4684111 |  |
|  | Marinomonas_posidonica_IVIA_Po_181_uid52545 | 0.0166 | 0.0508 | 3899854 |  |
|  | Psychrobacter_PRwf-1_uid15759 (*) | -0.0694 | -0.0263 | 752 | 800-2000 for Psychrobacter spp. (Rodriguez-Calleja *et al.* 2005) |
|  | Psychrobacter_arcticum_273-4_uid9633 (*) | -0.0388 | -0.0101 | 185 | 800-2000 for Psychrobacter spp. (Rodriguez-Calleja *et al.* 2005) |
|  | Psychrobacter_cryohalolentis_K5_uid13920 (*) | -0.0496 | -0.0227 | 1036 | 800-2000 for Psychrobacter spp. (Rodriguez-Calleja *et al.* 2005) |
|  | Pseudomonas_syringae_tomato_DC3000_uid359 (&) | 0.0164 | 0.0430 | 47 | 200-430 for Pseudomonas (Singh *et al.* 2006) |
|  | Thioflavicoccus_mobilis_8321_uid60883 | 0.0313 | 0.0237 |  |  |
|  | marine_bacterium_HP15_uid46089 | 0.0249 | 0.0848 | 3905679 |  |
| M_radiotolerans | Azorhizobium_caulinodans_ORS_571_uid19267 | -0.0106 | -0.0332 |  |  |
|  | Agromonas_oligotrophica_S58_uid191613 | -0.0279 | -0.0619 | 175 |  |
|  | Bradyrhizobium_BTAi1_uid16137 | -0.0183 | -0.0522 | 169384 |  |
|  | Bradyrhizobium_S23321_uid72425 | 0.0005 | -0.0358 | 7231219 |  |
|  | Beijerinckia_indica_ATCC_9039_uid20841 | -0.0295 | -0.0538 | 4049770 |  |
|  | Bradyrhizobium_japonicum_uid17 | -0.0200 | -0.0546 | 680784 |  |
|  | Brucella_suis_ATCC_23445_uid20371 (&) | 0.1490 | 0.1284 | 1810018 | 152 for Brucella abortus (Garcia *et al.* 1987) |
|  | Methylobacterium_4_46_uid18809 | -0.0523 | -0.0802 | 1153961 |  |
|  | Methylocystis_SC2_uid173412 | -0.0204 | -0.0430 | 2229694 |  |
|  | Methylobacterium_chloromethanicum_CM4_uid19527 | -0.0358 | -0.0602 | 8944 |  |
|  | Methylobacterium_extorquens_PA1_uid18637 (*) | -0.0337 | -0.0581 | 5470792 | 2700 (Nogueira *et al.* 1998) |
|  | Methylobacterium_nodulans_ORS_2060_uid20477 | -0.0497 | -0.0774 | 1349967 |  |
|  | Methylobacterium_populi_BJ001_uid19559 | -0.0277 | -0.0539 | 812 |  |
|  | Methylobacterium_radiotolerans_JCM_2831_uid18817 (*) | -0.0519 | -0.0785 | 4549678 | 2000 (Nogueira *et al.* 1998) |
|  | Nitrobacter_hamburgensis_X14_uid13473 | 0.0244 | -0.0136 | 120319 |  |
|  | Nitrobacter_winogradskyi_Nb-255_uid13474 | 0.0331 | -0.0049 | 114333 |  |
|  | Ochrobactrum_anthropi_ATCC_49188_uid19485 | 0.0883 | 0.0666 | 991 |  |
|  | Oligotropha_carboxidovorans_OM4_uid66839 | 0.0731 | 0.0340 | 3538916 |  |
|  | Rhodopseudomonas_palustris_BisA53_uid15751 | 0.0239 | -0.0147 | 422569 |  |
|  | Xanthobacter_autotrophicus_Py2_uid15756 | -0.0322 | -0.0554 | 2054010 |  |
| T_gammatolerans | Archaeoglobus_fulgidus_uid104 | -0.0121 | -0.0801 | 1429943 |  |
|  | Archaeoglobus_profundus_DSM_5631_uid32583 | -0.0147 | -0.0853 | 1303658 |  |
|  | Archaeoglobus_sulfaticallidus_PM70_1_uid196460 | 0.0066 | -0.0536 |  |  |
|  | Ferroglobus_placidus_DSM_10642_uid33635 | -0.0091 | -0.0826 |  |  |
|  | Methanocaldococcus_FS406_22_uid37943 | -0.0167 | -0.1050 | 1378754 |  |
|  | Methanocaldococcus_fervens_AG86_uid32615 | -0.0148 | -0.1018 | 970395 |  |
|  | Methanothermus_fervidus_DSM_2088_uid33689 | 0.0281 | -0.0230 |  |  |
|  | Methanotorris_igneus_Kol_5_uid51821 | -0.0041 | -0.0674 |  |  |
|  | Methanocaldococcus_infernus_ME_uid32611 | -0.0254 | -0.1101 | 298991 |  |
|  | Methanopyrus_kandleri_uid294 | 0.0173 | -0.0337 |  |  |
|  | Methanocaldococcus_vulcanius_M7_uid33047 | -0.0159 | -0.1046 | 39373 |  |
|  | Pyrococcus_NA2_uid65431 (*) | 0.0004 | -0.1360 | 579716 |  |
|  | Pyrococcus_ST04_uid162927 | -0.0123 | -0.1425 | 228332 |  |
|  | Pyrococcus_furiosus_COM1_uid163827 (*) | -0.0197 | -0.1559 | 1480209 | 3000 (DiRuggiero *et al.* 1997) |
|  | Pyrococcus_yayanosii_CH1_uid66055 | -0.0193 | -0.1571 | 1426784 |  |
|  | Thermococcus_4557_uid67883 | 0.0422 | -0.0970 | 1376025 |  |
|  | Thermococcus_CL1_uid167371 | 0.0162 | -0.1271 | 1020227 |  |
|  | Thermococcus_gammatolerans_EJ3_uid33671 (*) | -0.0217 | -0.1684 | 127165 | 6000 (Jolivet *et al.* 2003) |
|  | Thermococcus_litoralis_DSM_5473_uid81925 | 0.0325 | -0.1118 |  |  |
|  | Thermococcus_sibiricus_MM_739_uid34531 | 0.0501 | -0.0841 | 1783814 |  |
| H_salinarum | Halogeometricum_borinquense_DSM_11551_uid20743 |  | 0.0989 |  |  |
|  | Haloarcula_hispanica_N601_uid227070 |  | 0.0440 |  |  |
|  | Halorubrum_lacusprofundi_ATCC_49239_uid18455 |  | -0.0780 |  |  |
|  | Haloarcula_marismortui_ATCC_43049_uid105 |  | 0.0466 |  |  |
|  | Haloferax_mediterranei_ATCC_33500_uid43185 |  | 0.0777 |  |  |
|  | Halomicrobium_mukohataei_DSM_12286_uid27945 |  | 0.0137 |  |  |
|  | Halovivax_ruber_XH_70_uid59897 |  | 0.0341 |  |  |
|  | Halobacterium_salinarum_R1_uid106 (*) |  | 0.0877 |  |  |
|  | Haloterrigena_turkmenica_DSM_5511_uid30411 |  | 0.0494 |  |  |
|  | Halorhabdus_utahensis_DSM_12940_uid29305 |  | -0.0196 |  |  |
|  | Haloferax_volcanii_DS2_uid12524 |  | 0.0782 |  |  |
|  | Halopiger_xanaduensis_SH_6_uid56049 |  | 0.0701 |  |  |
|  | Natrinema_J7_uid89473 |  | 0.0448 |  |  |
|  | Natronobacterium_gregoryi_SP2_uid60135 |  | 0.0194 |  |  |
|  | Natrialba_magadii_ATCC_43099_uid30691 |  | 0.0782 |  |  |
|  | Natronococcus_occultus_SP4_uid46985 |  | 0.0748 |  |  |
|  | Natrinema_pellirubrum_DSM_15624_uid52951 |  | 0.0444 |  |  |
|  | Natronomonas_pharaonis_uid15742 |  | -0.0377 |  |  |
|  | Salinarchaeum_laminariae_Harcht_Bsk1_uid202311 |  | -0.0720 |  |  |
|  | halophilic_archaeon_DL31_uid52855 |  | -0.0162 |  |  |

**Table S3** Parameters *f_i_* and *p* and their 95% confidence intervals, for the model (1) fitted to the GOC_250_ vs. 16S rRNA data of different datasets. To improve readability, we report parameter *p* values as *a*, where, *a= -ln (p)* (and *p=e^-a^*). The confidence intervals for four parameter models were obtained in two steps – confidence interval for *f_i_* was estimated for the whole dataset and fixed at that estimation. This *f_i_* value was then used for the estimation of confidence intervals for the parameter *p* for the R-R, R-N and N-N categories of points. Therefore, confidence intervals for *f_i_* are the same for the two parameter and four parameter datasets.

| **Separate datasets (Two parameters per graph - all data on each graph pooled )** | | | | | | |
| --- | --- | --- | --- | --- | --- | --- |
| Dataset | *a*  (Parameter *p=e^-a^*) | Confidence interval of *a* (lower bound) | Confidence interval of *a* (higher bound) | *f_i_* | Confidence interval of *f_i_* (lower bound) | Confidence interval of *f_i_* (higher bound) |
| D_radiodurans_wholeDataset | 19.134 | 15.423 | 25.243 | 0.14 | 0.127 | 0.154 |
| E_faecium_wholeDataset | 31.963 | 24.863 | 44.929 | 0.322 | 0.298 | 0.343 |
| P_arcticum_wholeDataset | 14.488 | 11.764 | 19.795 | 0.172 | 0.137 | 0.204 |
| M_radiotolerans_wholeDataset | 46.217 | 40.647 | 53.132 | 0.186 | 0.175 | 0.201 |
| T_gammatolerans_wholeDataset | 94.066 | 78.261 | 114.74 | 0.152 | 0.147 | 0.158 |
| K_radiotolerans_wholeDataset | 24.269 | 20.935 | 27.993 | 0.248 | 0.214 | 0.276 |
| C_thermalis_wholeDataset | 53.05 | 44.451 | 67.22 | 0.091 | 0.084 | 0.098 |
| **Four parameter models (Parameter f_i_ same within dataset for R-R, R-N and N-N categories of points)** | | | | | | |
| Dataset | *a*  (Parameter *p=e^-a^*) | Confidence interval of *a* (lower bound) | Confidence interval of *a* (higher bound) | Parameter f_i_ taken from the two parameters model | Confidence interval of *f_i_* (lower bound) | Confidence interval of *f_i_* (higher bound) |
| D_radiodurans_R-R | 42.38 | 26.938 | 57.31 | 0.14 | 0.127 | 0.154 |
| D_radiodurans_R-N | 19.584 | 16.856 | 29.604 | 0.14 | 0.127 | 0.154 |
| D_radiodurans_N-N | 13.261 | 11.757 | 14.842 | 0.14 | 0.127 | 0.154 |
| E_faecium_R-R | 91.118 | N/A | N/A | 0.322 | N/A | N/A |
| E_faecium_R-N | 66.013 | 36.737 | 94.294 | 0.322 | 0.298 | 0.343 |
| E_faecium_N-N | 26.411 | 24.012 | 30.791 | 0.322 | 0.298 | 0.343 |
| P_arcticum_R-R | 32.994 | 31.805 | 33.48 | 0.172 | 0.137 | 0.204 |
| P_arcticum_R-N | 22.21 | 20.894 | 23.835 | 0.172 | 0.137 | 0.204 |
| P_arcticum_N-N | 12.886 | 12.085 | 14.03 | 0.172 | 0.137 | 0.204 |
| M_radiotolerans_R-R | 349.971 | N/A | N/A | 0.186 | N/A | N/A |
| M_radiotolerans_R-N | 60.582 | 40.534 | 639.143 | 0.186 | 0.175 | 0.201 |
| M_radiotolerans_N-N | 44.774 | 39.314 | 51.684 | 0.186 | 0.175 | 0.201 |
| T_gammatolerans_R-R | 93.983 | 61.488 | 206.467 | 0.152 | 0.147 | 0.158 |
| T_gammatolerans_R-N | 113.719 | 94.394 | 160.084 | 0.152 | 0.147 | 0.158 |
| T_gammatolerans_N-N | 82.1 | 63.66 | 105.735 | 0.152 | 0.147 | 0.158 |
| K_radiotolerans_R-R | 27.07 | 21.5 | 42.218 | 0.248 | 0.214 | 0.276 |
| K_radiotolerans_R-N | 26.503 | 23.587 | 29.561 | 0.248 | 0.214 | 0.276 |
| K_radiotolerans_N-N | 23.33 | 21.74 | 25.016 | 0.248 | 0.214 | 0.276 |
| C_thermalis_R-R | 135.846 | N/A | N/A | 0.091 | N/A | N/A |
| C_thermalis_R-N | 183.650 | N/A | N/A | 0.091 | N/A | N/A |
| C_thermalis_N-N | 53.033 | 45.608 | 64.872 | 0.091 | 0.084 | 0.098 |
| **Six parameters per dataset (R-R, R-N, and N-N categories of points fitted independently from each other)** | | | | | | |
| Dataset | *a*  (Parameter *p=e^-a^*) | Confidence interval of *a* (lower bound) | Confidence interval of *a* (higher bound) | *f_i_* | Confidence interval of *f_i_* (lower bound) | Confidence interval of *f_i_* (higher bound) |
| D_radiodurans_R-R | 52.596 | 21.634 | 541.001 | 0.155 | 0.13 | 0.194 |
| D_radiodurans_R-N | 17.229 | 14.288 | 27.652 | 0.136 | 0.129 | 0.142 |
| D_radiodurans_N-N | 12.71 | 8.874 | 17.535 | 0.125 | -0.012 | 0.198 |
| E_faecium_R-R | N/A | N/A | N/A | N/A | N/A | N/A |
| E_faecium_R-N | 51.714 | 20.447 | 79.264 | 0.295 | 0.263 | 0.347 |
| E_faecium_N-N | 25.139 | 20.745 | 34.901 | 0.314 | 0.288 | 0.343 |
| P_arcticum_R-R | 28.918 | 17.756 | 30.659 | 0.108 | -0.228 | 0.142 |
| P_arcticum_R-N | 20.094 | 18.606 | 22.084 | 0.163 | 0.157 | 0.17 |
| P_arcticum_N-N | 12.635 | 10.379 | 17.094 | 0.167 | 0.123 | 0.209 |
| M_radiotolerans_R-R | N/A | N/A | N/A | N/A | N/A | N/A |
| M_radiotolerans_R-N | 53.334 | 36.288 | 622.675 | 0.147 | 0.132 | 0.16 |
| M_radiotolerans_N-N | 46.179 | 40.362 | 54.064 | 0.198 | 0.184 | 0.215 |
| T_gammatolerans_R-R | 36.385 | 0.186 | 289.015 | -0.443 | -0.768 | 0.163 |
| T_gammatolerans_R-N | 113.457 | 93.255 | 163.895 | 0.152 | 0.143 | 0.163 |
| T_gammatolerans_N-N | 82.192 | 64.17 | 107.41 | 0.153 | 0.147 | 0.159 |
| K_radiotolerans_R-R | 18.176 | 15.154 | 44.918 | 0.135 | 0.082 | 0.257 |
| K_radiotolerans_R-N | 27.832 | 21.22 | 34.936 | 0.258 | 0.21 | 0.288 |
| K_radiotolerans_N-N | 24.004 | 19.522 | 29.209 | 0.256 | 0.201 | 0.296 |
| C_thermalis_R-R | N/A | N/A | N/A | N/A | N/A | N/A |
| C_thermalis_R-N | 319.642 | 23.484 | 356.331 | 0.088 | 0.078 | 0.098 |
| C_thermalis_N-N | 53.473 | 44.676 | 68.054 | 0.092 | 0.084 | 0.102 |

**
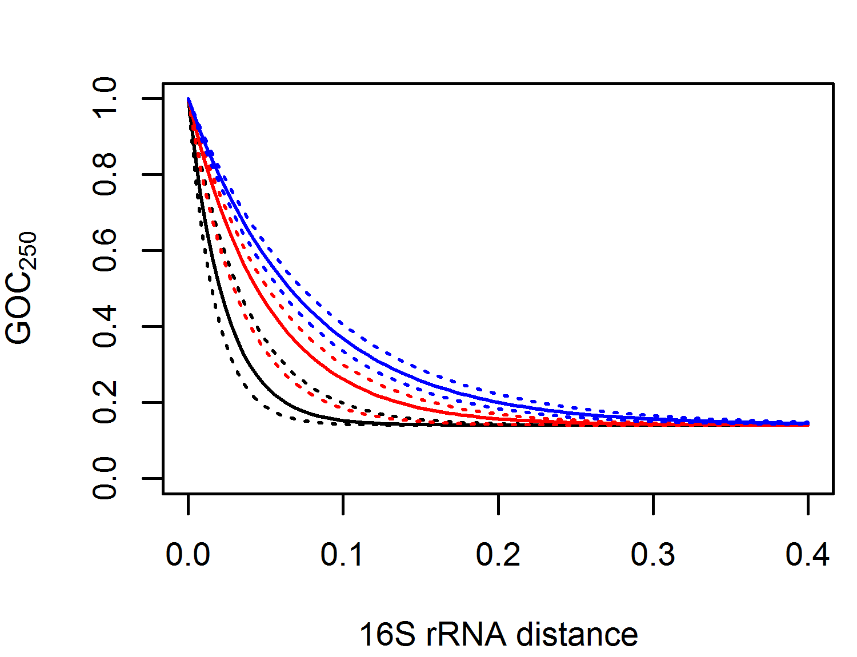
**

**Figure S1** 95% confidence intervals for parameter *p* for the model (1) fitted to the GOC_250_ vs. 16S rRNA data of the D_radiodurans dataset, given an *f_i_* value fixed at the whole dataset estimate (i.e. shown are confidence intervals for *p* presented in the four parameter model in Table S3). Parameter *p* estimates the rate of decline of the model fits to the GOC_250_-16S rRNA distance points. Shown are fits (full line) and confidence intervals (dotted lines) for three different categories of data points. Each data point represented a genome pair; the three categories of points were defined by radiation resistance (R) or nonresistance (N) of species in the species pair. Model describing the R-R category is shown in black, the R-N category in red, and the N-N category in blue.

**Supplementary literature cited (for Table S2)**

Albuquerque, L., C. Simoes, M. F. Nobre, N. M. Pino, J. R. Battista, *et al.*, 2005 *Truepera radiovictrix* gen. nov., sp. nov., a new radiation-resistant species and the proposal of Trueperaceae fam. nov. FEMS Microbiol. Lett. **247**: 161–169.

Anellis, A., D. Berkowitz, and D. Kemper, 1973 Comparative resistance of nonsporogenic bacteria to low-temperature gamma irradiation. Appl. Microbiol. **25**: 517–523.

Bagwell, C. E., S. Bhat, G. M. Hawkins, B. W. Smith, T. Biswas, *et al.*, 2008 Survival in nuclear waste, extreme resistance, and potential applications gleaned from the genome sequence of *Kineococcus radiotolerans* SRS30216. PLoS ONE **3**: e3878.

Battista, J. R., 1997 Against all odds: The survival strategies of *Deinococcus radiodurans*. Annu. Rev. Microbiol. **51**: 203–224.

Billi, D., E. I. Friedmann, K. G. Hofer, M. G. Caiola, and R. Ocampo-Friedmann, 2000 Ionizing-radiation resistance in the desiccation-tolerant cyanobacterium *Chroococcidiopsis*. Appl. Environ. Microbiol. **66**: 1489–1492.

Brandāo Areal, H., R. Charbonneau, and P. Dion, 1993 Effect of dose rate and comparison of different media used in the recuperation of gamma irradiated *Listeria monocytogenes*. Radiat. Phys. Chem. **42**: 655–658.

Daly, M. J., E. K. Gaidamakova, V. Y. Matrosova, A. Vasilenko, M. Zhai, *et al.*, 2004 Accumulation of Mn(II) in *Deinococcus radiodurans* facilitates gamma-radiation resistance. Science **306**: 1025–1028.

DiRuggiero, J., N. Santangelo, Z. Nackerdien, J. Ravel, and F. T. Robb, 1997 Repair of extensive ionizing-radiation DNA damage at 95 degrees C in the hyperthermophilic archaeon *Pyrococcus furiosus*. J. Bacteriol. **179**: 4643–4645.

Dulermo, R., S. Fochesato, L. Blanchard, and A. De Groot, 2009 Mutagenic lesion bypass and two functionally different RecA proteins in *Deinococcus deserti*. Mol. Microbiol. **74**: 194–208.

Ferreira, A. C., M. F. Nobre, F. A. Rainey, M. T. Silva, R. Wait, *et al.*, 1997 *Deinococcus geothermalis* sp. nov. and *Deinococcus murrayi* sp. nov., two extremely radiation-resistant and slightly thermophilic species from hot springs. Int. J. Syst. Bacteriol. **47**: 939–947.

Garcia, M. M., B. W. Brooks, R. B. Stewart, W. Dion, J. R. Trudel, *et al.*, 1987 Evaluation of gamma radiation levels for reducing pathogenic bacteria and fungi in animal sewage and laboratory effluents. Can. J. Vet. Res. **51**: 285–289.

Grant, I. R., and M. F. Patterson, 1992 Sensitivity of foodborne pathogens to irradiation in the components of a chilled ready meal. Food Microbiol. **9**: 95–103.

Gtari, M., I. Essoussi, R. Maaoui, H. Sghaier, R. Boujmil, *et al.*, 2012 Contrasted resistance of stone-dwelling *Geodermatophilaceae* species to stresses known to give rise to reactive oxygen species. FEMS Microbiol. Ecol. **80**: 566–577.

Jolivet, E., S. L’Haridon, E. Corre, P. Forterre, and D. Prieur, 2003 *Thermococcus gammatolerans* sp. nov., a hyperthermophilic archaeon from a deep-sea hydrothermal vent that resists ionizing radiation. Int. J. Syst. Evol. Microbiol. **53**: 847–851.

Kotiranta, A. K., H. Ito, M. P. Haapasalo, and K. Lounatmaa, 1999 Radiation sensitivity of *Bacillus cereus* with and without a crystalline surface protein layer. FEMS Microbiol. Lett. **179**: 275–280.

Makarova, K. S., M. V. Omelchenko, E. K. Gaidamakova, V. Y. Matrosova, A. Vasilenko, *et al.*., 2007 *Deinococcus geothermalis*: The pool of extreme radiation resistance genes shrinks. PLoS ONE **2**: e955.

Nishimura, Y., K. Uchida, K. Tanaka, T. Ino, and H. Ito, 1994 Radiation sensitivities of *Acinetobacter* strains isolated from clinical sources. J. Basic Microbiol. **34**: 357–360.

Nogueira, F., M. Luisa Botelho, and R. Tenreiro, 1998 Radioresistance studies in *Methylobacterium* spp. Radiat. Phys. Chem. **52**: 15–19.

Patterson, M., 1989 Sensitivity of *Listeria monocytogenes* to irradiation on poultry meat and in phosphate-buffered saline. Lett. Appl. Microbiol. **8**: 181–184.

Rodriguez, O., M. Castellperez, N. Ekpanyaskun, R. Moreira, and A. Castillo, 2006 Surrogates for validation of electron beam irradiation of foods. Int. J. Food Microbiol. **110**: 117–122.

Rodríguez-Calleja, J. M., M. F. Patterson, I. García-López, J. A. Santos, A. Otero, *et al.*, 2005 Incidence, radioresistance, and behavior of *Psychrobacter* spp. in rabbit meat. J. Food Prot. **68**: 538–543.

Saha, S. C., and B. A. Chopade, 2009 Radiation sensitivity of *Acinetobacter* spp. and their radicidation for preservation of meat at low temperature. Bangladesh Med. Res. Counc. Bull. **35**.

Saroj, S. D., R. Shashidhar, M. Pandey, V. Dhokane, S. Hajare, *et al.*, 2006 Effectiveness of radiation processing in elimination of *Salmonella typhimurium* and *Listeria monocytogenes* from sprouts. J. Food Prot. **69**: 1858–1864.

Shashidhar, R., S. A. Kumar, H. S. Misra, and J. R. Bandekar, 2010 Evaluation of the role of enzymatic and nonenzymatic antioxidant systems in the radiation resistance of *Deinococcus*. Can. J. Microbiol. **56**: 195–201.

Singh, R., P. Gupta, S. Purohit, P. Kumar, S. G. Vaijapurkar, *et al.*, 2006 Radiation resistance of the microflora associated with amniotic membranes. World J. Microbiol. Biotechnol. **22**: 23–27.

Yuan, M., W. Zhang, S. Dai, J. Wu, Y. Wang, *et al.*, 2009 *Deinococcus gobiensis* sp. nov., an extremely radiation-resistant bacterium. Int. J. Syst. Evol. Microbiol. **59**: 1513–1517.

Zhiping, W., Y. Qingfu, C. Hairui, X. Bujin, and W. Meiwen, 1998 Selection of the mutant of *Spirulina platensis* with extra-long filaments and morphology and growth of the mutant. Acta Agriculturae Nucleatae Sinica. **12**: 146-150.
